# Supplementary material for: A Molecular Electron Density Theory Study of the Synthesis of Spirobipyrazolines through the Domino Reaction of Nitrilimines with Allenoates
Source: Molecules. 2019 Nov 16;24(22):4159. doi: 10.3390/molecules24224159 (PMC6891705; doi:10.3390/molecules24224159)
Supplement: Supplementary file 1 [file molecules-24-04159-s001.pdf]

Supplementary Material

A Molecular Electron Density Theory Study of the Synthesis of Spirobipyrazolines through the Domino Reaction of Nitrilimines with Allenoates

Luis R. Domingo,[a] Fatemeh Ghodsi,[b] Mar Ríos-Gutiérrez[a,c]

a) Department of Organic Chemistry, University of Valencia, Dr Moliner 50, 46100 Burjassot, Valencia, Spain

b) Department of Chemistry, University of Sistan and Baluchestan, Zahedan, Iran

c) Department of Chemistry and Chemical Biology, McMaster University, 1280 Main Street West, Hamilton, Ontario L8S 4L8, Canada

Index

S2 Study of the reaction paths associated to the *cb-type* 32CA reaction of dimethyl NI **13** with the α,β-conjugated C4-C5 double bond of methyl 1-methyl-allenoate **4**.

S4 Table with the MPWB1K/6-311G(d) electronic energies, enthalpies, entropies and Gibbs free energies, computed at 25 ºC in DCM, of the stationary points involved in the domino reaction of diphenyl NI **1** with allenoate **4**.

S5 Table with the MPWB1K/6-311G(d) electronic energies, enthalpies, entropies and Gibbs free energies, computed at 25 ºC in DCM, of the stationary points involved in the *cb-type* 32CA reaction of dimethyl NI **13** with the α,β-conjugated C4-C5 double bond of methyl1-methyl-allenoate **4**.

*Study of the reaction paths associated to the cb-type 32CA reaction of dimethyl NI **13** with the α,β-conjugated C4-C5 double bond of methyl 1-methyl-allenoate **4**.*

The two regioisomeric reaction paths associated to the *cb-type* 32CA reaction between dimethyl NI **13** and the α,β-conjugated C4-C5 double bond of methyl 1-methyl-allenoate **4** were studied for a comparative analysis. They are related with the initial two-centre interaction between the most electrophilic centre of methyl 1-methyl-allenoate **4**, the β-conjugate C5 carbon, and the C1 carbon or the N3 nitrogen of dimethyl NI **13**. Relative enthalpies and Gibbs free energies in DCM are given in Scheme S1.

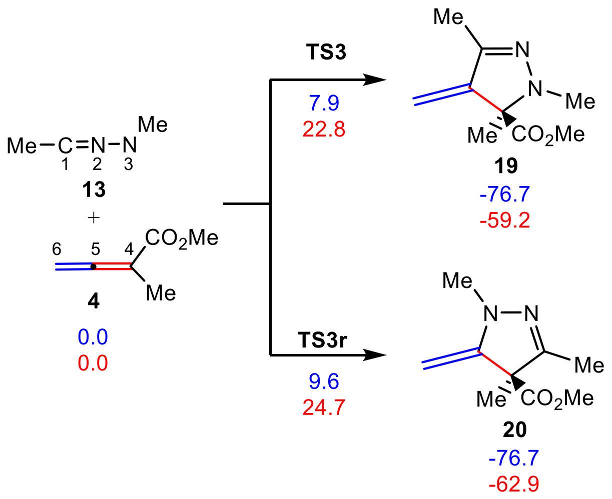

Scheme S1. Regioisomeric reaction paths associated with the *cb-type* 32CA reaction of dimethyl NI **13** with the α,β-conjugated C4-C5 double bond of methyl1-methyl-allenoate **4**. MPWB1K/6-311G(d) relative enthalpies, in blue, and Gibbs free energies, in red, are given in kcal mol^{-1}.

This *cb-type* 32CA reaction takes place through a one-step mechanism. Consequently, two regioisomeric TSs, **TS3** and **TS3r**, and two pyrazolines, **19** and **20**, were located and characterised. The activation enthalpies associated to the two competitive reaction paths are 7.9 (**TS3**) and 9.6 (**TS3r**) kcal mol^{-1}, the reaction beings strongly exothermic by 76.7 (**19**) and 76.7 (**20**) kcal mol^{-1}. Some appealing conclusions can be drawn from these energy results: i) the more favourable reaction path, which is associated with the formation of pyrazoline **19** via **TS3**, presents an activation enthalpy of 7.9 kcal mol^{-1}. This value is 2.7 kcal mol^{-1} lower than that associated with the 32CA reaction of diphenyl NI **1** with allenoate **4** (see Scheme 5). This reaction path is associated with the attack of the carbenoid C1 carbon of dimethyl NI **13** on the conjugated C5 carbon of allenoate **4**; ii) the attack of dimethyl NI **13** on the α,*β*-conjugated C4–C5 double bond of allenoate **4** is regioselective as **TS3r** is 1.7 kcal mol^{-1} above **TS3**; and iii) formation of

pyrazoline **19** is strongly exothermic by 76.7 kcal mol^{-1}. Consequently, this 32CA reaction can be considered irreversible.

The geometries of the TSs involved in the two competitive reaction paths associated with in the attack of dimethyl NI **13** on the α,β-conjugated C4–C5 double bond of allenoate **4** are given in Figure S1. At **TS3** the distances between the C1 and C5, and the N3 and the C4 centers at are 2.196 and 2.609 Å, while **TS3r** the distances between the N3 and C5, and the C1 and the C6 centers are 2.123 and 2.514 Å. Some appealing conclusions can be drawn from these geometrical parameters: i) the two TSs show a high asynchronicity; ii) in the two TSs, the short distance corresponds to the C1(N3)–C5 one, involving the β-conjugated C5 carbon of allenoate **4**; iii) considering that the C–C and C–N bond formation begins in the narrow range of distances of 2.0 - 1.9 and 1.9-1.8 Å, respectively, the distances between these interacting centers at these TSs indicate that formation of the C(N)-C single bond has not begun at any TSs; and finally iv) these distance at **TS3** are closer to those found at **TS11**, indicating that substitution of the two methyl groups in NI **13** for two phenyl groups In NI **1** does not produce any significant change in the TSs geometries.

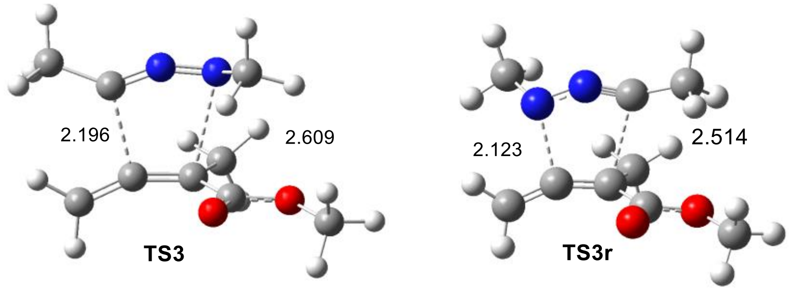

Figure S1. MPWB1K/6-311G(d) geometries in DCM of the two regioisomeric TSs involved in the 32CA reaction of dimethyl NI **13** with the α,β-conjugated C4-C5 double bond of methyl 1-methyl-allenoate **4**. Distances are given in Angstroms.

The polar character of this 32CA reaction was evaluated analysing the GEDT at the two regioisomeric TSs. Reactions with GEDT values of 0.0e correspond to non-polar processes, while values higher than 0.2e correspond to polar processes. The GEDT, which fluxes from the NI framework to the allenoate one is 0.21e at **TS3** and 0.21e at **TS3r**. These data indicate that this *cb-type* 32CA reaction has a polar character.

Table S1. MPWB1K/6-311G(d) Electronic energies (E, in a.u.), enthalpies (H, in a.u.), entropies (S, in cal mol^{-1} K^{-1}) and Gibbs free energies (G, in au), computed at 25 ℃ in DCM, of the stationary points involved in the domino reaction of diphenyl NI **1** with allenoate **4**.

	E	H	S	G
**1**	-610.633942	-610.420304	116.2	-610.475521
**4**	-383.748053	-383.607066	92.8	-383.651176
	-994.381995	-994.027370	209.1	-994.126697
**TS11**	-994.365799	-994.010482	152.4	-994.082911
**TS11r**	-994.360419	-994.005107	153.8	-994.078176
**TS21**	-994.355672	-994.000266	159.9	-994.076251
**TS21r**	-994.354445	-993.999362	158.5	-994.074673
**6**	-994.499070	-994.138652	148.1	-994.209023
**15**	-994.500514	-994.140017	148.4	-994.210512
**14**	-994.516074	-994.155136	147.8	-994.225380
**16**	-994.509506	-994.148330	148.7	-994.219002
**TS12**	-1605.121124	-1604.546407	208.7	-1604.645544
**TS12r**	-1605.117069	-1604.542144	207.1	-1604.640565
**TS22**	-1605.128913	-1604.553619	202.1	-1604.649659
**TS2r**	-1605.130346	-1604.555617	210.7	-1604.655747
**5**	-1605.229379	-1604.650092	202.4	-1604.746280
**17**	-1605.226631	-1604.646545	203.5	-1604.743227
**18**	-1605.229049	-1604.649476	205.8	-1604.747256

Table S2. MPWB1K/6-311G(d) Electronic energies (E, in a.u.), enthalpies (H, in a.u.), entropies (S, in cal mol^{-1} K^{-1}) and Gibbs free energies (G, in au), computed at 25 °C in DCM, of the stationary points involved in the *cb-type* 32CA reaction of dimethyl NI **13** with the α,β-conjugated C4-C5 double bond of methyl1-methyl-allenoate **4**.

	E	H	S	G
**4**	-383.748053	-383.607066	92.8	-383.651176
**13**	-227.248892	-227.150141	81.0	-227.188648
**TS3**	-610.985468	-610.744567	124.0	-610.803464
**TS3r**	-610.982578	-610.741854	123.3	-610.800447
**19**	-611.124942	-610.879418	115.4	-610.934244
**20**	-611.129451	-610.88403	117.9	-610.940026
